# Optimizing Fatigue Performance in Gradient Structural Steels by Manipulating the Grain Size Gradient Rate

**DOI:** 10.3390/ma17133210

**Published:** 2024-07-01

**Authors:** Meichen Pan, Xin Chen, Meiling He, Yi Kong, Yong Du, Alexander Hartmaier, Xiaoyu Zheng, Yuling Liu

**Affiliations:** 1 State Key Laboratory of Powder Metallurgy, Central South University, Changsha 410083, China; panmeichen18012105@163.com (M.P.); 213307007@csu.edu.cn (X.C.); annelhml@163.com (M.H.); yikong@csu.edu.cn (Y.K.); yong-du@csu.edu.cn (Y.D.); 2 Interdisciplinary Centre for Advanced Materials Simulation (ICAMS), Ruhr-Universität Bochum, 44801 Bochum, Germany; alexander.hartmaier@ruhr-uni-bochum.de

**Keywords:** gradient structural steel, gradient rate, fatigue, crack propagation

## Abstract

As a new type of high-performance material, gradient structural steel is widely used in engineering fields due to its unique microstructure and excellent mechanical properties. For the prevalent fatigue failure problem, the rate of change in the local grain size gradients along the structure (referred to as the gradient rate) is a key parameter in the design of gradient structures, which significantly affects the fatigue performance of gradient structural steel. In this study, a new method of ‘Voronoi primary + secondary modeling’ is adopted to successfully establish three typical high-strength steel models corresponding to the convex-, linear-, and concave-type gradient rates for gradient structures, focusing on the stress–strain response and crack propagation in structural steel with different gradient rates under cyclic loading. It was found that the concave gradient rate structural model is dominated by finer grains with larger volume fraction, which is conducive to hindering fatigue crack propagation and has the longest fatigue life, which is 16.16% longer than that of the linear gradient rate structure and 23.66% longer than that of the convex gradient rate structure. The simulation results in this study are consistent with the relevant experimental phenomena. Therefore, when regulating the gradient rate, priority should be given to increasing the volume fraction of fine grains and designing a gradient rate structure dominated by fine grains to improve the fatigue life of the material. This study presents a new strategy for designing engineering materials with better service performance.

## 1. Introduction

With the development of modern industrial technology, people are increasingly demanding increased performance from engineering materials, and gradient structural steel, a new type of high-performance material due to its unique microstructure and excellent mechanical properties, has attracted widespread attention in the scientific research community [1,2]. Its internal grain size from one side to the other is a continuous change, forming unique ‘gradient’ characteristics [3,4,5]; this characteristic directly affects the strength, plastic toughness, and fatigue properties of the material [6,7,8,9]. Among them, the grain size gradient is one of the most common types of gradient-structured metallic materials [10,11].

According to statistics, more than 90% of metallic engineering materials fail due to fatigue [12,13]. Generated by surface induction [14,15], shot peening [16,17,18], heat treatment [19], mechanical grinding [20], and pre-deformation [21] can form gradient layers on material surfaces, with thicknesses ranging from hundreds of nanometers to several millimeters. These surface gradient layers effectively reduce the incidence of surface damage, suppressing the failure behavior of materials and enhancing their fatigue properties. Since Fang et al. [6] first reported the preparation of gradient nano-grained (GNG) Cu through surface mechanical grinding treatment (SMGT), a series of related studies has shown that gradient structures enable materials to exhibit better combinations of strength and toughness [22,23], as well as improved fatigue resistance [24,25] and wear resistance [26]. Ding et al. [27] prepared grain size gradient Inconel 718 alloy using SMGT. Jiang et al. [5] prepared grain size gradient Inconel 718 alloy through ultrasonic surface rolling treatment (USRP). Theoretical models such as the low-cycle fatigue crack propagation model [28], and finite element simulation studies such as the results of Tilbrook [29], Zeng [23], Wang [30], Luo [25], and others, reveal that gradient materials play an important role in crack propagation behavior, fatigue fracture characteristics, etc. Li and Soh [31] simulated the strengthening effect of samples containing tens of nanometers to tens of micrometers of grains by using the finite element method. Wu et al. [26] studied the deformation mechanism of gradient materials, and found that the grain size gradient under uniaxial stretching produces a macroscopic strain gradient due to the evolution of incompatible deformation along the depth of the gradient, which transforms the applied uniaxial stress into a multiaxial stress, leading to a large number of dislocations accumulating and interacting with each other, and generating additional strain hardening.

In particular, the rate of change in the local grain size gradients along the structure (referred to as the gradient rate) is an important parameter describing the trend in grain size change [10], and in the process of gradient structure construction, different processing methods and different degrees of treatment can significantly affect the gradient rate of a structure. The concept of gradient rate makes it possible to quantitatively describe the relationship between the gradient structure and the mechanical properties of materials, and also lays the foundation for a deeper understanding of the mechanisms related to gradient-structured metallic materials. As a key parameter in the design of gradient structures, the gradient rate delicately balances the distribution pattern of strength and toughness; therefore, it is hoped that the gradient rate in the design of gradient structures can be used to more accurately control the fatigue crack initiation and extension, thus enhancing the reliability and durability of the materials under long-term service conditions.

To date, most research has focused on the toughening properties of gradient materials [32,33,34,35], ignoring their effect on fatigue properties, and there is a lack of research on the critical factors that influence the performance of gradient structures [36]. Lin et al. [35] prepared pure Ni samples with gradient structures by electrodeposition technique and precisely controlled the grain size gradient to obtain the optimal grain size distribution, resulting in yield strength and uniform elongation superior to the coarse crystalline Ni. However, the effects of different gradient rates on the fatigue properties have not thoroughly been investigated. Wang et al. [30] demonstrated that the grain size gradient structure could improve the yield strength of the material without reducing its flexibility, but the difference brought by the different degrees of variation in the grain gradient was not taken into account.

The above studies have shown that the grain size gradient rate in gradient structures significantly affects their mechanical properties and deformation mechanisms. Although existing studies have revealed the positive effect of gradient structure on fatigue performance, there is still insufficient understanding of how the gradient rate precisely regulates fatigue life, crack initiation threshold, and cyclic deformation behavior, which undoubtedly constitutes a technological bottleneck, restricting the further optimization and industrialization of its application [37,38]. On the other hand, the preparation of controllable gradient rate structures is still difficult in experiments [35], and the intrinsic connection between grain size gradient rate and fatigue performance has not yet been clarified; is there an optimal gradient rate structure? The type of grain size gradient rate for obtaining optimal fatigue performance is still not fully understood and requires further investigation.

With no sophisticated methods for controlling gradient rates, researchers urgently need to conduct more systematic and in-depth research to reveal the intrinsic law between the gradient rate and the key properties of gradient structural steel through the establishment of an accurate gradient rate structural model for numerical study, to realize the precise control of the gradient structure, establish accurate gradient rate structure models, explore efficient and economical preparation processes to achieve precise control of gradient structure [39], and to provide theoretical support for the future experimental aspects of the study.

Therefore, this study establishes three types of models of gradient structural steel under different gradient rates and simulates the stress–strain response and crack propagation behavior under fatigue loading by means of the finite element method. It is for the sake of systematically investigating the intrinsic connection between the gradient rate and the fatigue performance of gradient structural steel, further revealing the regulation strategy for the strength–toughness of gradient structural materials, and providing theoretical support for achieving the engineering of gradient structural materials for directional and the improvement of fatigue life of high-strength structural steel.

## 2. Materials and Methods

### 2.1. Three Types of Gradient Rate Polycrystalline Models

This study focuses on a typical high-strength structural steel (Chinese grade Q690, 0.12C-2.24Mn-0.26Si-0.07Cr-0.05Nb-0.02Ni) with a yield strength of 690 MPa, tensile strength of 734 MPa, and fracture elongation of 15.4%. With high strength and toughness, Q690 is widely used in the bridge and construction industry, but its fatigue failure problem is a key challenge in the engineering field. Based on the uniaxial tensile test of Q690 steel [40], we can obtain the true stress–strain data, as shown in Table 1.

**Figure 1:**
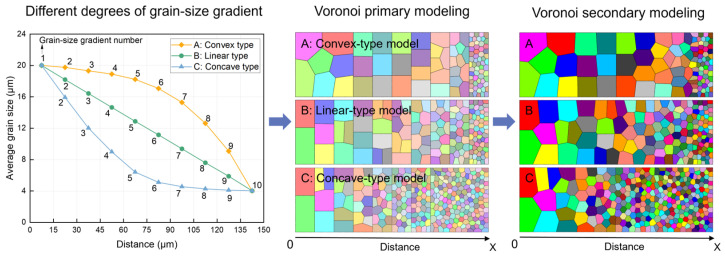
Schematic diagram of the modeling process of gradient polycrystalline models with the three grain size gradient rates, and the different colors in the modes indicate different grains.

Typically, gradient rates consist of three types, namely, convex (A), linear (B), and concave (C). Figure 1 shows a schematic of the position of the three types of grain size gradients along the horizontal X direction. The maximum and minimum grain sizes remain constant under different circumstances, while the distribution of grain sizes varies greatly. Among them, the grain size change in gradient A is first flat and then steep, resulting in a larger volume fraction of coarse grains. The grain size change in gradient B is approximately constant, and the distribution of coarse-grained and fine-grained grains is relatively uniform. The grain size change in gradient C is steep and then flat, resulting in a greater volume fraction of fine grains (i.e., hard phase).

In this study, the “Voronoi primary + secondary modeling” method was used to construct a gradient polycrystalline geometry model with different gradient rates, using Neper open-source software (Version 4.9.0) [41]. Firstly, ten data points were taken on each of the three types of gradient rate curves, and ten equal intervals (15*k* × 50*k*, scale bar *k* = actual structure size/model size, and *k* is a non-zero constant) were used for comparison in the simulated region (150*k* × 50*k*). The number of grains in each interval was converted according to the gradient rate in Figure 1, and then the coordinates of the gradient polycrystalline seed centroid of ten intervals were output, which is called the Voronoi primary modeling process. Next, the centroid coordinate file was used as a new input file, and Neper’s Voronoi algorithm was used to model the whole region to obtain a two-dimensional gradient polycrystalline geometry model with a continuous gradient change in grain size, which is called the Voronoi secondary modeling process.

**Figure 2:**
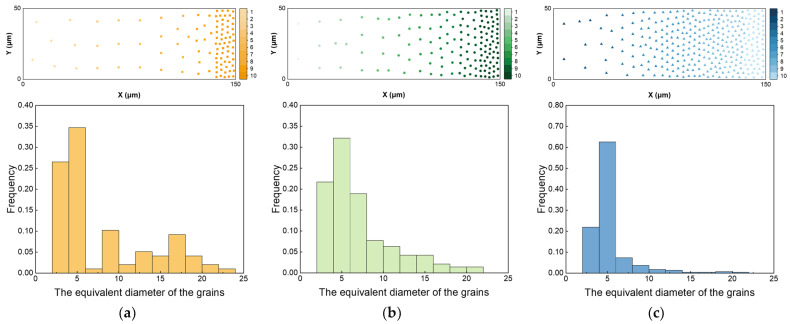
Schematic diagram of random distribution and grain size distribution in gradient polycrystalline seed intervals: (**a**) grain seed distribution (top) and particle size distribution (bottom) in the convex gradient rate model; (**b**) grain seed distribution (top) and particle size distribution (bottom) in the linear gradient rate model; (**c**) grain seed distribution (top) and particle size distribution (bottom) in the concave gradient rate model.

Through the above method, three types of gradient polycrystalline models corresponding to different gradient rates can be constructed, as shown in Figure 1; the two-dimensional gradient structure geometry model along the X and Y directions are 150 μm and 50 μm. The total grain numbers of the three types of gradient models are 98, 143, and 302, respectively, and the grain size varies from 20 μm to 4 μm. In addition, the meshing type is quadrilateral, the length of the grid edge is 0.5, and the total number of grids is 30,000. Figure 2 shows the distribution of gradient polycrystalline seeds (top) and the equivalent grain size (bottom) for the three types of gradient models.

### 2.2. Elastoplastic and Damage Constitutive Equation

For the three types of gradient models, we need to specify the elastic and hardening behavior of the gradient grains, respectively. In the present study, the modulus of elasticity was 207.0 GPa, the Poisson’s ratio was 0.3, and the density of steel is 7.8 g/cm^3^.

In addition, we reasonably set the average size and yield strength of all grains in the model following the Hall–Petch equation [30,42]:

(1) $$\sigma_{y}=\sigma_{y0}+k_{y}d^{-\frac{1}{2}}$$

where *σ*_y_ is the yield strength of the material with an average grain size of *d*, *σ*_y0_ is the constant related to the material itself, and *k*_y_ is the Hall–Petch coefficient (here, the value of 632.456 MPa·μm^1/2^ [42]). In this study, when *σ*_y_ = 690.00 MPa, *σ*_y0_ = 548.58 MPa. From this, *σ*_y0_ and *σ*_y_ for different average grain sizes in the model can be calculated. Note that the term “average grain size” refers to the local values of grain sizes in the gradient microstructure. The regions in which these local grain sizes are evaluated are indicated by the numbers in Figure 1.

To simplify the model, and further assume that the tensile strength of the material is also dependent on the grain size, the tensile strength of the individual grains is assigned by a specific equation [30,42]:

(2) $$\sigma_{\mathrm{ts}}=\sigma_{\mathrm{ts}0}+k_{y}d^{-\frac{1}{2}}$$

where *σ*_ts_ is the tensile strength, *σ*_ts0_ is the constant related to the material itself, *k*_y_ is the Hall–Petch coefficient, and *d* is the average grain size. Similar to Equation (1), the *σ*_ts0_ and *σ*_ts_ corresponding to the different average grain sizes in the model can be obtained.

Combined with the parameters listed in Equations (1) and (2) above, the engineering tensile stress–strain curves of three types of gradient rate structures (Figure 2a–c) can be obtained.

**Figure 3:**
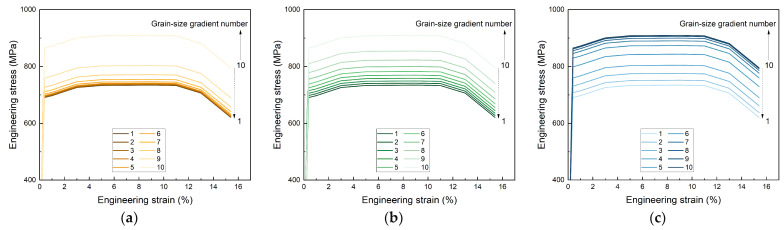

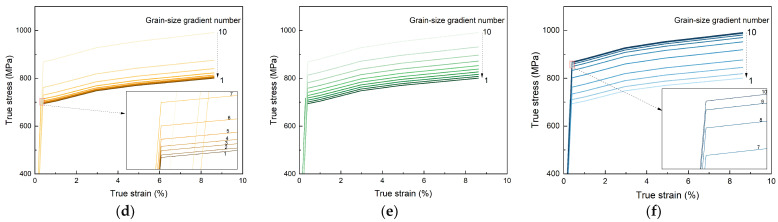
Engineering stress–strain curves and true stress–strain curves of materials: (**a**) the engineering stress–strain curves of materials in the convex gradient rate model; (**b**) the engineering stress–strain curves of materials in the linear gradient rate model; (**c**) the engineering stress–strain curves of materials in the concave gradient rate model; (**d**) the true stress–strain curves of materials in the convex gradient rate model; (**e**) the true stress–strain curves of materials in the linear gradient rate model; (**f**) the true stress–strain curves of materials in the concave gradient rate model.

These engineering stress–strain curves are converted to true stress–strain curves by Equations (3) and (4) [43]:

(3) $$\sigma_{\mathrm{true}}=\sigma_{\mathrm{eng}}(\varepsilon_{\mathrm{eng}}+1)$$

(4) $$\varepsilon_{\mathrm{true}}=\ln(\varepsilon_{\mathrm{eng}}+1)$$

where *σ*_eng_ and *ε*_eng_ are engineering stress and engineering strain, and *σ*_true_ and *ε*_true_ are true stress and true strain. Then, engineering stress–strain curves (Figure 3a–c) and true stress–strain curves (Figure 3d–f) of materials can be obtained, and each sub-figure contains ten separate curves, some of which are very close to each other. The global stress–strain values (Tables S1 and S2 in the Supplementary Materials) have been obtained by calculating the yield strength and tensile strength of different grains based on the Hall–Petch equation, thus assigning different material parameters to different gradient grains according to grain size.

**Figure 4:**
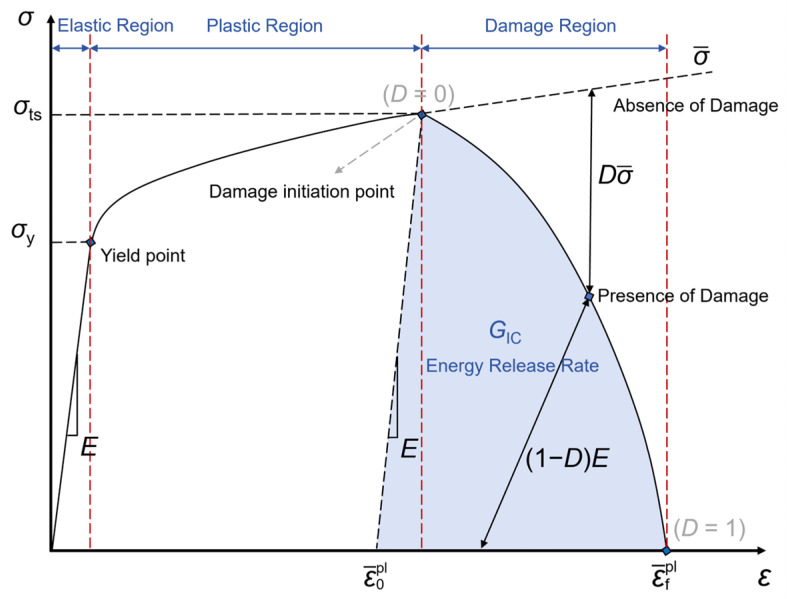
Stress–strain curves with progressive damage degradation.

In order to describe the material degradation during plastic deformation, a damage model was employed in this study. For the sake of generality, this study chose the ductile damage evolution model embedded in Abaqus software (Version 2020) as the damage constitutive model of the material. The damage evolution of elastoplastic materials includes the reduction in the flow stress and the degradation of elasticity, and the characteristic stress–strain behavior of the ductile metal subjected to damage is shown in Figure 4, where the solid black line represents the damaged stress–strain curve and the dashed line above the solid line represents the curve without damage. In Figure 4, *E* is the elastic modulus of the material, *σ*_y_ is the yield strength of the material, *σ*_ts_ is the tensile strength of the material and the stress of the material when it is damaged, ${\bar{\varepsilon }}_{0}^{\mathrm{pl}}$is the equivalent plastic strain at the time of damage, and ${\bar{\varepsilon }}_{f}^{\mathrm{pl}}$ is the equivalent plastic strain at failure, i.e., when the overall damage variable reaches *D* = 1 [44]. The damage constitutive model specifies at least four ductile damage parameters, including stress triaxiality, fracture strain, strain rate, and fracture energy. In this study, the triaxiality of stress was taken as 0.33, the strain rate was taken as 0.00, and the formula for calculating the fracture energy was as follows [44]:

*G*_f_ = *G*_IC_ × *l* (5)

where *G*_f_ is the fracture energy, *G*_IC_ is the area of the shaded blue part in Figure 4, the physical meaning is the critical energy release rate, *l* is the characteristic edge length of the element, and for the 2D model, and *l* is the edge length of the mesh, i.e., *l* = 0.5. The main ductile damage parameters (the fracture strain and fracture energy) for three types of gradient rate structures are shown in Table S3.

### 2.3. Pre-Processing and Abaqus/Explicit Calculation Method

**Figure 5:**
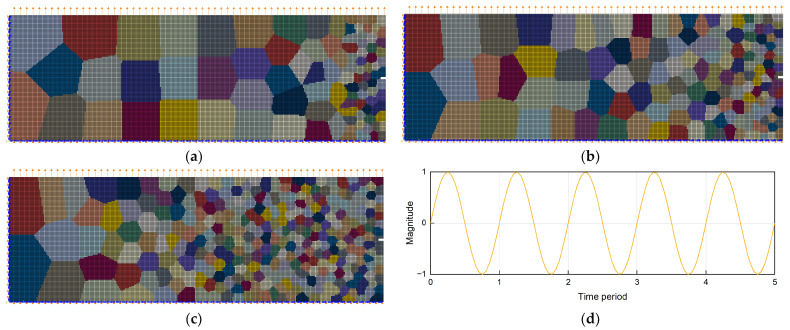
Schematic diagram of the model after applying boundary conditions and cyclic loads in Abaqus software (Version 2020): (**a**) the convex gradient rate model; (**b**) the linear gradient rate model; (**c**) the concave gradient rate model; (**d**) schematic diagram of the cyclic load amplitude curve applied to the model. The different colors in the figure indicate different grains, the arrows on the top surface indicate the direction of displacement loading, the arrows at the bottom and left side indicate that the boundary conditions of the model are fully fixed, and the right side of the model is prefabricated with a microcrack measuring 2 μm × 1 μm.

The gradient models shown in Figure 1 were imported into the commercial finite element package Abaqus (Version 2020) in a pre-processing step. To simulate the conditions of uniaxial fatigue, suited boundary conditions were applied, where the nodes at the bottom and the left side have been fixed and the load is applied to the nodes on the top surface of the model, as shown in Figure 5a–c. The initial load amplitude was set to 500.00 MPa, which was converted into a displacement-controlled loading amplitude of 0.12 μm, resulting in a tension–compression amplitude ratio of R = −1; Figure 5d shows a schematic diagram of the load amplitude. The nodes on the right-hand side, where the smallest grains are situated, are free of boundary conditions, mimicking the free surface of the material. Since the initial crack in the actual microstructure usually originates in this fine-grained region of the surface of the material, an initial microcrack is prefabricated at the center of the right end of the model to study the crack propagation behavior in the different microstructures. The size of prefabricated microcracks is 2 μm × 1 μm. By comparing the fatigue crack propagation behavior in different gradient rate models, the optimal strategy to design a gradient rate for maximum fatigue performance of gradient materials was derived.

Based on the established gradient structure model under different gradient rates, display analysis solver was employed in Abaqus software (Version 2020). The single cycle length of the models was 1 s, and the total loading time set for the simulation was 200 s. At the same time, using reasonable quality scaling (quality scaling factor set to 1000 and target time increment set to 0.0001) and time scaling (64-core parallel computation), a single case could be completed in 10–15 min to efficiently analyze the influence of gradient rate on the stress–strain response and fatigue crack propagation behavior.

## 3. Results

**Figure 6:**
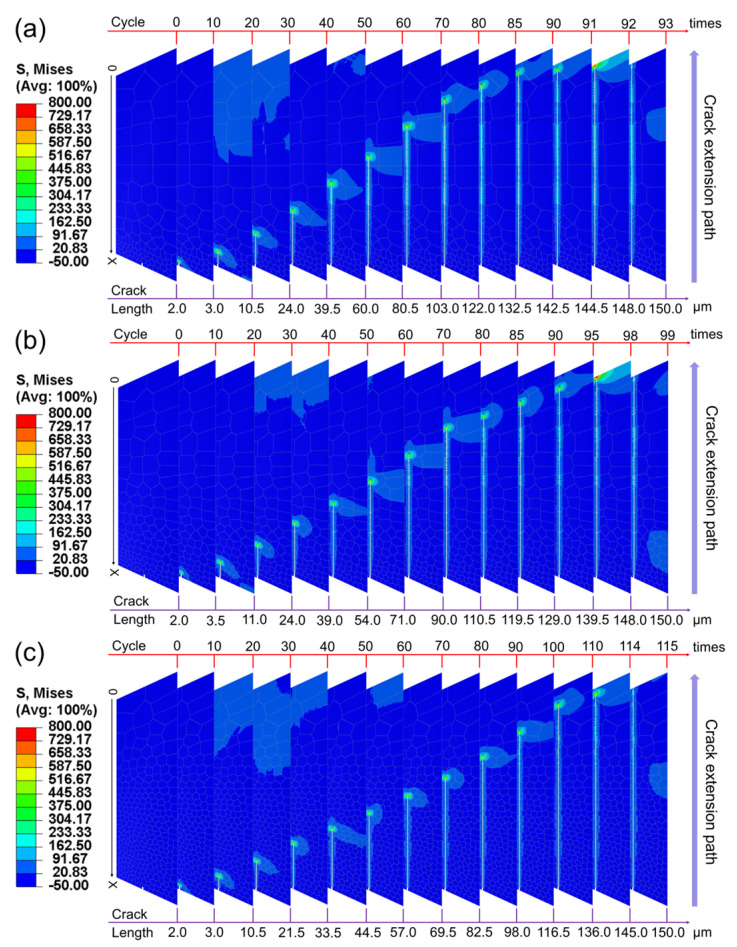
Mises stress results under cyclic loading for the three types of gradient rate models: (**a**) the convex gradient rate model; (**b**) the linear gradient rate model; (**c**) the concave gradient rate model.

**Figure 7:**
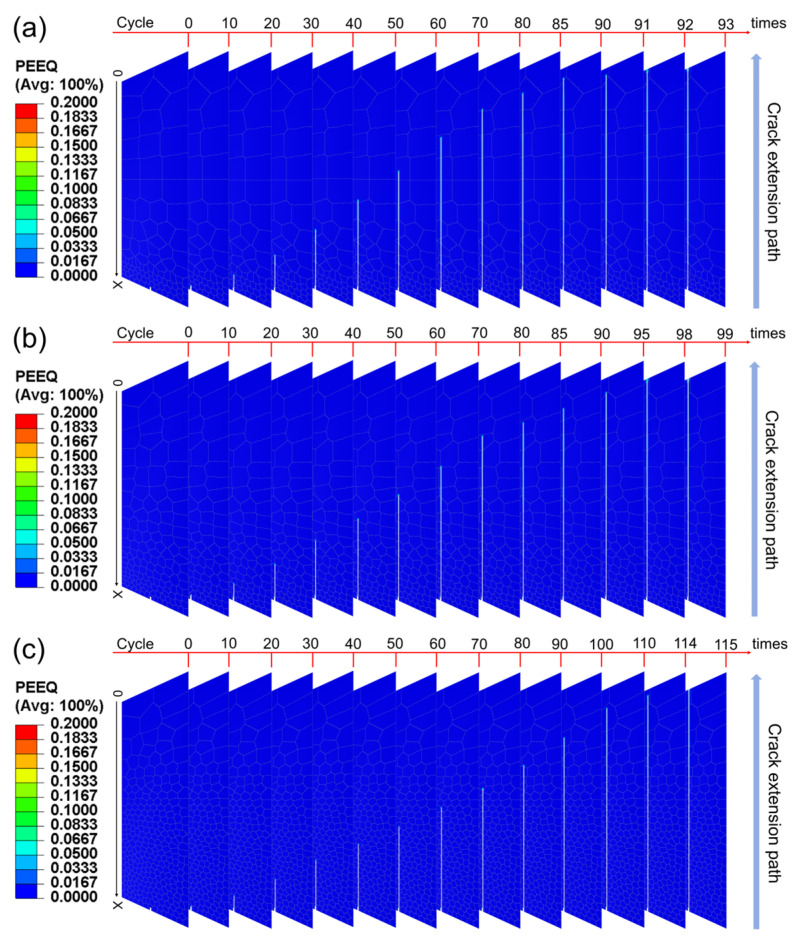
Equivalent plastic strain results under cyclic loading for three types of gradient rate models: (**a**) the convex gradient rate model; (**b**) the linear gradient rate model; (**c**) the concave gradient rate model.

By performing finite element simulations under cyclic loading on the gradient structure of high-strength steel models corresponding to three types of gradient rates, the Mises stress values and equivalent plastic strain values under different loading cycles and the crack propagation length were obtained. Figure 6 and Figure 7 show the Mises stress and equivalent plastic strain (PEEQ) result contours for different gradient models, respectively, and the cycle numbers of different stages and their crack propagation lengths are marked in the diagrams to visually demonstrate the crack propagation process. The Mises stress varies from −50 MPa to 800 MPa in the contours with a more obvious change, and the equivalent plastic strain in the contours varies from 0 to 0.2 with a less obvious change.

## 4. Discussion

As can be seen from Figure 6 and Figure 7, as the number of load cycles increases, the crack propagates from the initial position in the direction perpendicular to the normal stress until a certain critical point is reached, and the crack completely penetrates the entire model, indicating that the material has completely failed. It is worth noting that even at the same cycle, the length of crack propagation in each gradient rate model shows significant differences. This phenomenon reveals the direct effect of the non-uniform variation of grain size and its spatial distribution on the crack propagation rate in the gradient structure. Hanlon et al. [45] investigated the effect of grain size on the fatigue response of nanocrystalline metals and found that an increase in grain size usually leads to a decrease in the fatigue endurance limit due to mechanisms such as periodic deflection of the fatigue crack path at grain boundaries during crystallographic fracture. In this study, the gradient distribution of grain size not only determines the mechanical properties of the local microzone, but also regulates the stress field and strain energy release path at the crack tip at the relatively macroscopic level, significantly affecting the dynamic behavior of crack propagation.

**Figure 8:**
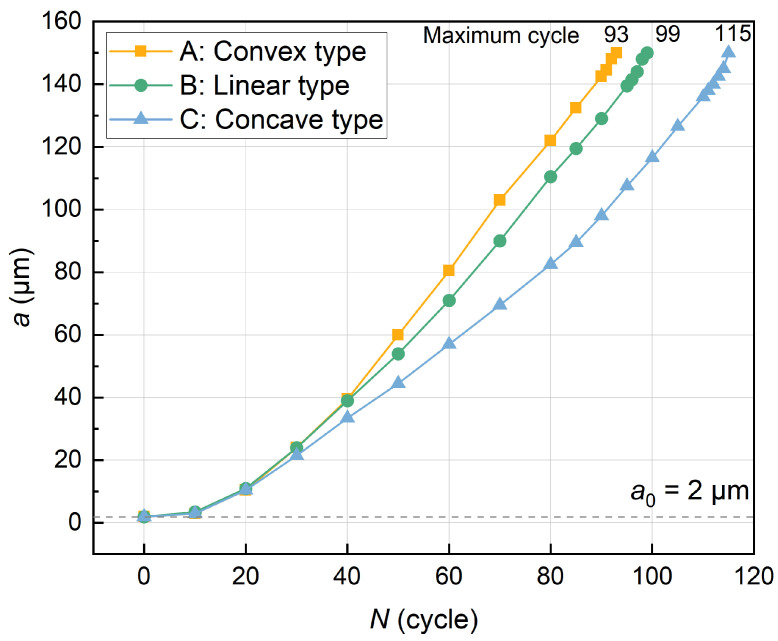
Fatigue life curves of three types of gradient models under cyclic loads: curve A belongs to the convex gradient rate model, curve B belongs to the linear gradient rate model, and curve C belongs to the concave gradient rate model.

In order to further reveal the quantitative relationship between crack propagation and the number of cycles, a graph of the variation in crack propagation length with the number of cycles was drawn based on the simulated data, as shown in Figure 8.

Figure 8 clearly shows the significant differences in fatigue life between the three types of gradient structures. The C-type (concave type) gradient structure showed the most superior fatigue durability, with the slowest crack propagation rate and the longest fatigue life, while the B-type (linear type) structure was the second, and the A-type (convex type) structure had the shortest fatigue life. It is reasonable to infer that the C-type gradient structure has a stronger effect in inhibiting fatigue crack propagation.

In-depth comparison of the A-type and C-type gradient structures shows that although the grain size of the coarsest grains and the finest grains are the same, the difference in gradient rate will still cause a large difference in the uniaxial tensile mechanical properties of the gradient structure materials. The coarse grains in the A-type structure have a relatively high integration number and show relatively weak yield strength and excellent plastic deformation ability. The C-type structure is dominated by fine grains with large volume fractions and shows stronger yield strength. This comparison reveals the importance of the fine grain volume fraction in the grain size gradient structure to regulate the fatigue life of high-strength steels. Wang et al. [10] also specified that fine grains can withstand higher stress loading and coarse grains can carry more plastic deformation. Specifically, the wide presence and uniform distribution of fine grains in the C-type gradient structure can effectively hinder the initiation and propagation of fatigue cracks, and delay the energy accumulation and release process at the crack front by increasing the tortuosity of the crack path, enhancing the dislocation interaction and improving the local plastic deformation ability, thereby significantly improving the fatigue life of the material. Conversely, A-type structures, although they have a higher potential for plastic deformation, result in a lower yield strength and the shortest fatigue life due to the high volume fraction of their coarse grains.

As for the B-type structure, the gradient rate of fine and coarse grains in the structure changes less, the grain size change transition is more uniform, and the volume fraction of coarse and fine grains is uniform; therefore, the plasticity of the structure remains unchanged while increasing the strength. Compared with A-type structures with poor performance and C-type structures with high preparation difficulty, the B-type gradient structure can take into account the performance and preparation cost to a certain extent.

Next, the propagation behavior of fatigue cracks is further analyzed according to the Paris equation [46], which establishes the relationship between the stress intensity factor and crack propagation rate. It is the basis for predicting the fatigue crack propagation life theory in today’s engineering applications, and it takes the following form:

d*a*/d*N* = *C* (∆*K*)*^m^* (6)

where *a* is the crack length, *N* is the number of stress cycles, d*a*/d*N* is the crack propagation rate, ∆*K* is the amplitude of the stress intensity factor, *C* and *m* are the material constants, and environmental factors such as temperature, humidity, medium, loading frequency, etc., are implicit in the constants, which can be obtained by fitting the experimental data.

The stress intensity factor, *K*, and the stress intensity factor amplitude, ∆*K*, are expressed as [47]:

(7) $$K=f\sigma \sqrt{\pi a\;}$$

(8) $$∆K=K_{\max}-K_{\min}=f\sigma_{\max}\sqrt{\pi a\;}-f\sigma_{\min}\sqrt{\pi a}=∆f\sigma \sqrt{\pi a\;}$$

where *σ*_max_ and *σ*_min_ are the maximum stress and minimum stress, respectively, and the difference between the two is the stress amplitude, ∆*σ*; *f* is the correction coefficient related to the geometry and size of the crack body, the load mode and the boundary conditions, etc., for the central crack wide plate *f* = 1, for the unilateral crack wide plate *f* = 1.12 [47].

Taking the common logarithm on both sides of Equation (6), the relationship between the fatigue crack propagation rate, d*a*/d*N*, and the amplitude of the stress intensity factor, ∆*K*, can be obtained as follows:

lg(d*a*/d*N) = m*lg(∆*K*) *+* lg*C* (9)

**Figure 9:**
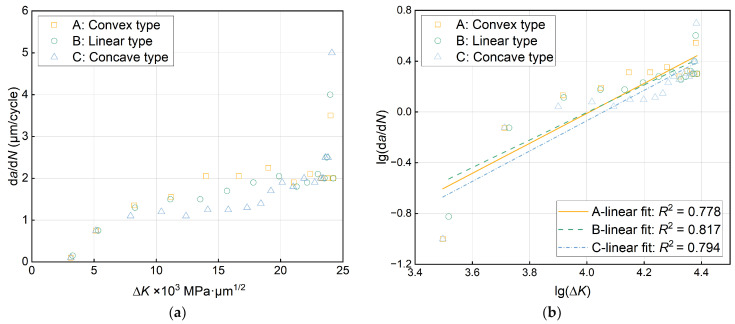
Fatigue data scatter and fitting results: (**a**) data scatter of fatigue crack propagation rate, d*a*/d*N*, and stress intensity factor amplitude, ∆*K*; (**b**) data scatter and linear fitting results of double logarithmic lg(d*a*/d*N*) and lg(∆*K*).

However, the data obtained from the fatigue crack propagation simulation are the crack length, *a*, and the number of cycles, *N*, so the appropriate data processing method must be used to calculate (d*a*/d*N*)*_i_* and the corresponding (∆*K*)*_i_*. The double logarithmic coordinates were used for regression fitting; then, the lg(d*a*/d*N*) − lg(∆*K*) relationship curve and the *m* and *C* values of the material constant were obtained. The secant method used in this study is a simple and fast method for processing data, which is suitable for calculating the slope of a straight line connecting two adjacent data points on an *a*–*N* curve, which is calculated as [47]:

(10) $${\left(da/dN\right)}_{\bar{a}}=\left(a_{i+1}-a_{i}\right)/\left(N_{i+1}-N_{i}\right)$$

where ${\left(da/dN\right)}_{\overset{-}{a}}$ is the average rate of the increment (*a_i_*_+1_ − *a_i_*), so it is necessary to calculate the value of (∆*K*)*_i_* by substituting the average crack length, $\bar{a}=\left(a_{i+1}+a_{i}\right)/2$, into Equation (8). The derived (d*a*/d*N*)*_i_* and the corresponding (∆*K*)*_i_* are plotted as shown in Figure 9a,b, showing the three sets of data points and their results after linear fitting based on the calculations in Equations (6)–(10); the Paris equation for fatigue crack propagation for the three types of gradient rate models is summarized in Table 2.

**Figure 10:**
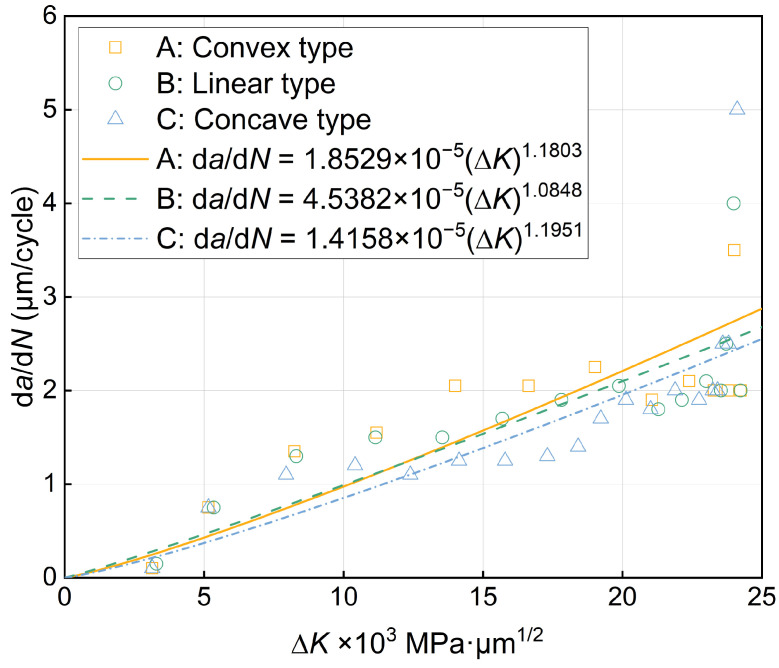
Fitting curves of fatigue crack propagation rate, d*a*/d*N*, and stress intensity factor amplitude, ∆*K*.

From the fitting results of Figure 10, it can be seen that the crack propagation rate of A-type structures is lower than that of B-type structures in the initial stage, and when a certain critical value is reached, the crack propagation rate completely exceeds that of the B-type structure, while the crack propagation rate of type structure has always maintained the lowest crack propagation rate in this simulation due to the wide distribution of fine grains and its effective suppression of crack propagation, which verifies its high efficiency in inhibiting fatigue damage. As for whether there is a critical value between the B-type structure and the C-type structure, it is not possible to determine the value in this simulation, and it is necessary to further study the influence of coarse-grained integration numbers and fine-grained size to obtain the optimal critical coarse-grained grain volume fraction and critical fine-grained grain size.

Experiments have shown that different gradient distributions correspond to different proportions of fine grains (hard phase) and coarse grains (soft phase), and the interactions between the layers result in completely different mechanical properties [48,49,50,51,52,53]. Huang et al. [49] experimentally found that compared with the coarse-grained (CG) samples, the fatigue strength of SMRT (surface mechanical rolling treatment) samples was significantly enhanced in both low-cycle and high-cycle fatigue states, and the fatigue strength increased by 33% compared to that of the CG sample. Wang et al. [50] prepared gradient layers that showed a reduction in surface layer strength from 1600 MPa to about 400 MPa, with a corresponding increase in tensile ductility from 2% to 13%. Yang et al. [53] investigated the effect of gradient changes in surface grain size and grain size along depth on the fatigue life of metallic materials and found that the rate of short crack extension decreases with the decreasing surface grain size and grain size gradient. In this study, the maximum fatigue life of the C-type gradient structure was increased by 23.66% compared with the A-type gradient structure, and 16.16% compared with the B-type structure. The C-type structure exhibits higher yield strength and tensile strength, while the change in plastic strain is smaller, which is the main reason for improved fatigue properties.

## 5. Conclusions

In this study, three models of high-strength steels with different types of gradient microstructures have been investigated under cyclic loading to quantify the effect of different gradient rates on the fatigue properties of the material. The corresponding types of microstructures gradients different in the gradient rate changed, where convex-, linear-, and concave-type gradient rate models have been established. Comparing the stress–strain response and crack propagation in different gradient rate models, it was found that the concave gradient structure model is dominated by fine grains with a larger volume fraction, which is conducive to hindering the propagation of fatigue cracks and results in the longest fatigue life. In contrast, the convex-type structure has a high plastic deformation potential, but the coarse grain volume fraction is higher, resulting in a lower yield strength and the shortest fatigue life. The linear gradient structure has a better match between strength and plasticity, and the fatigue life is in between the other cases. The maximum fatigue life of the concave gradient rate structure is 16.16% longer than that of the linear gradient rate structure and 23.66% longer than that of the convex gradient rate structure. When designing and controlling the gradient rate, priority should be given to increasing the volume fraction of fine grains, i.e., selecting a concave gradient rate structure dominated by fine grains, to obtain structural materials with longer fatigue life. The computational simulation results in this study are consistent with the relevant experimental phenomena, and further show that structural materials with better fatigue properties can be obtained by adjusting the gradient rate in the gradient structure.

The findings of this study not only deepen the understanding of the fatigue mechanism of gradient structural materials, but also provide ideas and strategies for the design of engineering materials with higher durability, especially in those application scenarios that need to withstand cyclic loading for a long time, such as aerospace, bridge construction, and energy facilities.

Admittedly, the present study has some limitations, such as the lack of direct experimental validation, which is due to the difficulty in preparing samples with different gradient rates. In addition, future research directions include the quantitative relationship between gradient rate and fatigue life of gradient structures, the prediction of fatigue life based on gradient structures, and so on.

## Figures and Tables

**Table 1:** The true stress–strain data of Q690 steel (based on the uniaxial tensile test [40]).

Point	1	2	3	4	5	6	7	8
Strain	0.000	0.003	0.010	0.020	0.030	0.049	0.068	0.086
Stress (MPa)	0.000	692.070	704.324	726.046	747.687	770.007	785.220	800.551

**Table 2:** Fitting results of three grouped data points based on the secant method.

Model	*m*	lg*C*	*C*	*R* ^2^
A	1.1803	−4.7322	1.8529 × 10^−5^	0.778
B	1.0848	−4.3431	4.5382 × 10^−5^	0.817
C	1.1951	−4.8490	1.4158 × 10^−5^	0.794

## Data Availability

The raw data supporting the conclusions of this article will be made available by the authors on request.

## Supplementary Materials

The following supporting information can be downloaded at: https://www.mdpi.com/article/10.3390/ma17133210/s1, Table S1: Engineering stress–strain data for three types of gradient rate models; Table S2: True stress–strain data for three types of gradient rate models; Table S3: The ductile damage parameters for three types of gradient rate models.
