# Supplementary material for: Optimizing Fatigue Performance in Gradient Structural Steels by Manipulating the Grain Size Gradient Rate
Source: Materials (Basel). 2024 Jul 1;17(13):3210. doi: 10.3390/ma17133210 (PMC11242507; doi:10.3390/ma17133210)
Supplement: Supplementary file 1 [file materials-17-03210-s001.zip › materials-3056853-supplementary.pdf]

Supplementary Materials

Optimizing Fatigue Performance in Gradient Structural Steels by Manipulating the Grain Size Gradient Rate

Meichen Pan, Xin Chen, Meiling He, Yi Kong, Yong Du, Alexander Hartmaier, Xiaoyu Zheng, and Yuling Liu

Table S1. Engineering stress-strain data for three-types of gradient rate models

**Gradient number**	**1**		**2**		**3**	
	Strain	Stress (MPa)	Strain	Stress (MPa)	Strain	Stress (MPa)
A (Convex type)	0.000	0.000	0.000	0.000	0.000	0.000
	0.003	690.000	0.003	690.892	0.003	692.505
	0.010	697.350	0.010	698.242	0.010	699.855
	0.020	711.810	0.020	712.702	0.020	714.315
	0.030	725.910	0.030	726.802	0.030	728.415
	0.050	733.340	0.050	734.232	0.050	735.845
	0.070	733.850	0.070	734.742	0.070	736.355
	0.090	734.450	0.090	735.342	0.090	736.955
	0.110	732.950	0.110	733.842	0.110	735.455
	0.130	706.230	0.130	707.122	0.130	708.735
	0.154	620.810	0.154	621.702	0.154	623.315
	Strain	Stress (MPa)	Strain	Stress (MPa)	Strain	Stress (MPa)
B (Linear type)	0.000	0.000	0.000	0.000	0.000	0.000
	0.003	690.000	0.003	696.869	0.003	704.657
	0.010	697.350	0.010	704.219	0.010	712.007
	0.020	711.810	0.020	718.679	0.020	726.467
	0.030	725.910	0.030	732.779	0.030	740.567
	0.050	733.340	0.050	740.209	0.050	747.997
	0.070	733.850	0.070	740.719	0.070	748.507
	0.090	734.450	0.090	741.319	0.090	749.107
	0.110	732.950	0.110	739.819	0.110	747.607
	0.130	706.230	0.130	713.099	0.130	720.887
	0.154	620.810	0.154	627.679	0.154	635.467
	Strain	Stress (MPa)	Strain	Stress (MPa)	Strain	Stress (MPa)
C (Concave type)	0.000	0.000	0.000	0.000	0.000	0.000
	0.003	690.000	0.003	707.089	0.004	731.153
	0.010	697.350	0.010	714.439	0.010	738.503
	0.020	711.810	0.020	728.899	0.020	752.963
	0.030	725.910	0.030	742.999	0.030	767.063
	0.050	733.340	0.050	750.429	0.050	774.493
	0.070	733.850	0.070	750.939	0.070	775.003
	0.090	734.450	0.090	751.539	0.090	775.603
	0.110	732.950	0.110	750.039	0.110	774.103
	0.130	706.230	0.130	723.319	0.130	747.383
	0.154	620.810	0.154	637.899	0.154	661.963

(continue)

Gradient number	4		5		6	
	Strain	Stress (MPa)	Strain	Stress (MPa)	Strain	Stress (MPa)
	0.000	0.000	0.000	0.000	0.000	0.000
	0.003	694.096	0.003	696.747	0.003	701.702
	0.010	701.446	0.010	704.097	0.010	709.052
	0.020	715.906	0.020	718.557	0.020	723.512
	0.030	730.006	0.030	732.657	0.030	737.612
A (Convex type)	0.050	737.436	0.050	740.087	0.050	745.042
	0.070	737.946	0.070	740.597	0.070	745.552
	0.090	738.546	0.090	741.197	0.090	746.152
	0.110	737.046	0.110	739.697	0.110	744.652
	0.130	710.326	0.130	712.977	0.130	717.932
	0.154	624.906	0.154	627.557	0.154	632.512
	Strain	Stress (MPa)	Strain	Stress (MPa)	Strain	Stress (MPa)
	0.000	0.000	0.000	0.000	0.000	0.000
	0.003	713.874	0.004	724.942	0.004	738.069
	0.010	721.224	0.010	732.292	0.010	745.419
	0.020	735.684	0.020	746.752	0.020	759.879
	0.030	749.784	0.030	760.852	0.030	773.979
B (Linear type)	0.050	757.214	0.050	768.282	0.050	781.409
	0.070	757.724	0.070	768.792	0.070	781.919
	0.090	758.324	0.090	769.392	0.090	782.519
	0.110	756.824	0.110	767.892	0.110	781.019
	0.130	730.104	0.130	741.172	0.130	754.299
	0.154	644.684	0.154	655.752	0.154	668.879
	Strain	Stress (MPa)	Strain	Stress (MPa)	Strain	Stress (MPa)
	0.000	0.000	0.000	0.000	0.000	0.000
	0.004	759.749	0.004	798.774	0.004	829.186
	0.010	767.099	0.010	806.124	0.010	836.536
	0.020	781.559	0.020	820.584	0.020	850.996
	0.030	795.659	0.030	834.684	0.030	865.096
C (Concave type)	0.050	803.089	0.050	842.114	0.050	872.526
	0.070	803.599	0.070	842.624	0.070	873.036
	0.090	804.199	0.090	843.224	0.090	873.636
	0.110	802.699	0.110	841.724	0.110	872.136
	0.130	775.979	0.130	815.004	0.130	845.416
	0.154	690.559	0.154	729.584	0.154	759.996

(continue)

Gradient number	7		8		9	
	Strain	Stress (MPa)	Strain	Stress (MPa)	Strain	Stress (MPa)
A (Convex type)	0.000	0.000	0.000	0.000	0.000	0.000
	0.003	710.375	0.004	726.674	0.004	758.698
	0.010	717.725	0.010	734.024	0.010	766.048
	0.020	732.185	0.020	748.484	0.020	780.508
	0.030	746.285	0.030	762.584	0.030	794.608
	0.050	753.715	0.050	770.014	0.050	802.038
	0.070	754.225	0.070	770.524	0.070	802.548
	0.090	754.825	0.090	771.124	0.090	803.148
	0.110	753.325	0.110	769.624	0.110	801.648
	0.130	726.605	0.130	742.904	0.130	774.928
	0.154	641.185	0.154	657.484	0.154	689.508
	Strain	Stress (MPa)	Strain	Stress (MPa)	Strain	Stress (MPa)
B (Linear type)	0.000	0.000	0.000	0.000	0.000	0.000
	0.004	755.303	0.004	778.297	0.004	809.844
	0.010	762.653	0.010	785.647	0.010	817.194
	0.020	777.113	0.020	800.107	0.020	831.654
	0.030	791.213	0.030	814.207	0.030	845.754
	0.050	798.643	0.050	821.637	0.050	853.184
	0.070	799.153	0.070	822.147	0.070	853.694
	0.090	799.753	0.090	822.747	0.090	854.294
	0.110	798.253	0.110	821.247	0.110	852.794
	0.130	771.533	0.130	794.527	0.130	826.074
	0.154	686.113	0.154	709.107	0.154	740.654
	Strain	Stress (MPa)	Strain	Stress (MPa)	Strain	Stress (MPa)
C (Concave type)	0.000	0.000	0.000	0.000	0.000	0.000
	0.004	845.732	0.004	855.365	0.004	861.691
	0.010	853.082	0.010	862.715	0.010	869.041
	0.020	867.542	0.020	877.175	0.020	883.501
	0.030	881.642	0.030	891.275	0.030	897.601
	0.050	889.072	0.050	898.705	0.050	905.031
	0.070	889.582	0.070	899.215	0.070	905.541
	0.090	890.182	0.090	899.815	0.090	906.141
	0.110	888.682	0.110	898.315	0.110	904.641
	0.130	861.962	0.130	871.595	0.130	877.921
	0.154	776.542	0.154	786.175	0.154	792.501

(continue)

Gradient number	10	
	Strain	Stress (MPa)
A (Convex type)	0.000	0.000
	0.004	864.807
	0.010	872.157
	0.020	886.617
	0.030	900.717
	0.050	908.147
	0.070	908.657
	0.090	909.257
	0.110	907.757
	0.130	881.037
	0.154	795.617
	Strain	Stress (MPa)
B (Linear type)	0.000	0.000
	0.004	864.807
	0.010	872.157
	0.020	886.617
	0.030	900.717
	0.050	908.147
	0.070	908.657
	0.090	909.257
	0.110	907.757
	0.130	881.037
	0.154	795.617
	Strain	Stress (MPa)
C (Concave type)	0.000	0.000
	0.004	864.807
	0.010	872.157
	0.020	886.617
	0.030	900.717
	0.050	908.147
	0.070	908.657
	0.090	909.257
	0.110	907.757
	0.130	881.037
	0.154	795.617

Table S2. True stress-strain data for three-types of gradient rate models

**Gradient number**	**1**		**2**		**3**	
	Strain	Stress (MPa)	Strain	Stress (MPa)	Strain	Stress (MPa)
	0.000	0.000	0.000	0.000	0.000	0.000
	0.003	692.070	0.003	692.965	0.003	694.583
	0.010	704.324	0.010	705.224	0.010	706.854
A (Convex type)	0.020	726.046	0.020	726.956	0.020	728.601
	0.030	747.687	0.030	748.606	0.030	750.267
	0.049	770.007	0.049	770.944	0.049	772.637
	0.068	785.220	0.068	786.174	0.068	787.900
	0.086	800.551	0.086	801.523	0.086	803.281
	Strain	Stress (MPa)	Strain	Stress (MPa)	Strain	Stress (MPa)
	0.000	0.000	0.000	0.000	0.000	0.000
	0.003	692.070	0.003	698.960	0.003	706.771
	0.010	704.324	0.010	711.261	0.010	719.127
B (Linear type)	0.020	726.046	0.020	733.053	0.020	740.996
	0.030	747.687	0.030	754.762	0.030	762.784
	0.049	770.007	0.049	777.219	0.049	785.397
	0.068	785.220	0.068	792.569	0.068	800.902
	0.086	800.551	0.086	808.038	0.086	816.527
	Strain	Stress (MPa)	Strain	Stress (MPa)	Strain	Stress (MPa)
	0.000	0.000	0.000	0.000	0.000	0.000
	0.003	692.070	0.003	709.210	0.004	734.078
	0.010	704.324	0.010	721.583	0.010	745.888
C (Concave type)	0.020	726.046	0.020	743.477	0.020	768.022
	0.030	747.687	0.030	765.289	0.030	790.075
	0.049	770.007	0.049	787.950	0.049	813.218
	0.068	785.220	0.068	803.505	0.068	829.253
	0.086	800.551	0.086	819.178	0.086	845.407

(continue)

**Gradient number**	**4**		**5**		**6**	
	Strain	Stress (MPa)	Strain	Stress (MPa)	Strain	Stress (MPa)
	0.000	0.000	0.000	0.000	0.000	0.000
	0.003	696.178	0.003	698.837	0.003	703.807
	0.010	708.460	0.010	711.138	0.010	716.143
A (Convex type)	0.020	730.224	0.020	732.928	0.020	737.982
	0.030	751.906	0.030	754.637	0.030	759.740
	0.049	774.308	0.049	777.091	0.049	782.294
	0.068	789.602	0.068	792.439	0.068	797.741
	0.086	805.015	0.086	807.905	0.086	813.306
	Strain	Stress (MPa)	Strain	Stress (MPa)	Strain	Stress (MPa)
	0.000	0.000	0.000	0.000	0.000	0.000
	0.003	716.016	0.004	727.842	0.004	741.021
	0.010	728.436	0.010	739.615	0.010	752.873
B (Linear type)	0.020	750.398	0.020	761.687	0.020	775.077
	0.030	772.278	0.030	783.678	0.030	797.198
	0.049	795.075	0.049	806.696	0.049	820.479
	0.068	810.765	0.068	822.607	0.068	836.653
	0.086	826.573	0.086	838.637	0.086	852.946
	Strain	Stress (MPa)	Strain	Stress (MPa)	Strain	Stress (MPa)
	0.000	0.000	0.000	0.000	0.000	0.000
	0.004	762.788	0.004	801.969	0.004	832.503
	0.010	774.770	0.010	814.185	0.010	844.901
C (Concave type)	0.020	797.190	0.020	836.996	0.020	868.016
	0.030	819.529	0.030	859.725	0.030	891.049
	0.049	843.243	0.049	884.220	0.049	916.152
	0.068	859.851	0.068	901.608	0.068	934.149
	0.086	876.577	0.086	919.114	0.086	952.263

(continue)

Gradient number	7		8		9	
	Strain	Stress (MPa)	Strain	Stress (MPa)	Strain	Stress (MPa)
	0.000	0.000	0.000	0.000	0.000	0.000
	0.003	712.506	0.004	729.581	0.004	761.733
	0.010	724.902	0.010	741.364	0.010	773.708
A (Convex type)	0.020	746.829	0.020	763.454	0.020	796.118
	0.030	768.674	0.030	785.462	0.030	818.446
	0.049	791.401	0.049	808.515	0.049	842.140
	0.068	807.021	0.068	824.461	0.068	858.726
	0.086	822.759	0.086	840.525	0.086	875.431
	Strain	Stress (MPa)	Strain	Stress (MPa)	Strain	Stress (MPa)
	0.000	0.000	0.000	0.000	0.000	0.000
	0.004	758.324	0.004	781.410	0.004	813.083
	0.010	770.280	0.010	793.503	0.010	825.366
B (Linear type)	0.020	792.655	0.020	816.109	0.020	848.287
	0.030	814.949	0.030	838.633	0.030	871.127
	0.049	838.575	0.049	862.719	0.049	895.843
	0.068	855.094	0.068	879.697	0.068	913.453
	0.086	871.731	0.086	896.794	0.086	931.180
	Strain	Stress (MPa)	Strain	Stress (MPa)	Strain	Stress (MPa)
	0.000	0.000	0.000	0.000	0.000	0.000
	0.004	849.115	0.004	858.786	0.004	865.138
	0.010	861.613	0.010	871.342	0.010	877.731
C (Concave type)	0.020	884.893	0.020	894.719	0.020	901.171
	0.030	908.091	0.030	918.013	0.030	924.529
	0.049	933.526	0.049	943.640	0.049	950.283
	0.068	951.853	0.068	962.160	0.068	968.929
	0.086	970.298	0.086	980.798	0.086	987.694

(continue)

Gradient number	10	
	Strain	Stress (MPa)
	0.000	0.000
	0.004	868.266
	0.010	880.879
A (Convex type)	0.020	904.349
	0.030	927.739
	0.049	953.554
	0.068	972.263
	0.086	991.090
	Strain	Stress (MPa)
	0.000	0.000
	0.004	868.266
	0.010	880.879
B (Linear type)	0.020	904.349
	0.030	927.739
	0.049	953.554
	0.068	972.263
	0.086	991.090
	Strain	Stress (MPa)
	0.000	0.000
	0.004	868.266
	0.010	880.879
C (Concave type)	0.020	904.349
	0.030	927.739
	0.049	953.554
	0.068	972.263
	0.086	991.090

Table S3. The ductile damage parameters for three-types of gradient rate models

**Gradient number**		**1**	**2**	**3**	**4**	**5**
A (Convex type)	Fracture strain	0.0865	0.0864	0.0864	0.0864	0.0864
	Fracture energy (MPa)	23.1466	23.1767	23.2312	23.285	23.3745
B (Linear type)	Fracture strain	0.0865	0.0864	0.0864	0.0863	0.0863
	Fracture energy (MPa)	23.1466	23.3787	23.6419	23.9536	24.3282
C (Concave type)	Fracture strain	0.0865	0.0864	0.0863	0.0861	0.0859
	Fracture energy (MPa)	23.1466	23.7241	24.5386	25.5082	26.8346

(continue)

**Gradient number**		**6**	**7**	**8**	**9**	**10**
A (Convex type)	Fracture strain	0.0864	0.0864	0.0863	0.0861	0.0856
	Fracture energy (MPa)	23.542	23.8353	24.3869	25.4725	29.0875
B (Linear type)	Fracture strain	0.0862	0.0861	0.086	0.0859	0.0856
	Fracture energy (MPa)	24.7729	25.3573	26.1382	27.2116	29.0875
C (Concave type)	Fracture strain	0.0858	0.0857	0.0857	0.0856	0.0856
	Fracture energy (MPa)	27.8709	28.4356	28.7647	28.9809	29.0875
